# The impact of polyphenols on chondrocyte growth and survival: a preliminary report

**DOI:** 10.3402/fnr.v59.29311

**Published:** 2015-10-05

**Authors:** Salvador Fernández-Arroyo, Fernando Huete-Toral, María Jesús Pérez de Lara, María de la Luz Cádiz-Gurrea, Laurence Legeai-Mallet, Vicente Micol, Antonio Segura-Carretero, Jorge Joven, Jesús Pintor

**Affiliations:** 1 Unitat de Recerca Biomèdica, Hospital Universitari de Sant Joan, IISPV, Universitat Rovira i Virgili, Reus, Spain; 2 Campus of International Excellence Southern Catalonia, Tarragona, Spain; 3 Department of Biochemistry, Faculty of Optics and Optometry, Universidad Complutense de Madrid, Madrid, Spain; 4 Functional Food Research and Development Center, Health Science Technological Park, Granada, Spain; 5 Department of Analytical Chemistry, University of Granada, Granada, Spain; 6 Imagine Institute – INSERM U1163, Necker Hospital for Sick Children, Paris, France; 7 Institute of Molecular and Cell Biology, Miguel Hernández University, Elche, Spain

**Keywords:** chondrodysplasia, fibroblast growth factor, *Hibiscus sabdariffa*, nutrition, osteoarthritis, polyphenols, signaling pathways

## Abstract

**Background:**

Imbalances in the functional binding of fibroblast growth factors (FGFs) to their receptors (FGFRs) have consequences for cell proliferation and differentiation that in chondrocytes may lead to degraded cartilage. The toxic, proinflammatory, and oxidative response of cytokines and FGFs can be mitigated by dietary polyphenols.

**Objective:**

We explored the possible effects of polyphenols in the management of osteoarticular diseases using a model based on the transduction of a mutated human FGFR3 (G380R) in murine chondrocytes. This mutation is present in most cases of skeletal dysplasia and is responsible for the overexpression of FGFR3 that, in the presence of its ligand, FGF9, results in toxic effects leading to altered cellular growth.

**Design:**

Different combinations of dietary polyphenols derived from plant extracts were assayed in FGFR3 (G380R) mutated murine chondrocytes, exploring cell survival, chloride efflux, extracellular matrix (ECM) generation, and grade of activation of mitogen-activated protein kinases.

**Results:**

Bioactive compounds from *Hibiscus sabdariffa* reversed the toxic effects of FGF9 and restored normal growth, suggesting a probable translation to clinical requests in humans. Indeed, these compounds activated the intracellular chloride efflux, increased ECM generation, and stimulated cell proliferation. The inhibition of mitogen-activated protein kinase phosphorylation was interpreted as the main mechanism governing these beneficial effects.

**Conclusions:**

These findings support the rationale behind the encouragement of the development of drugs that repress the overexpression of FGFRs and suggest the dietary incorporation of supplementary nutrients in the management of degraded cartilage.

The available evidence no longer sustains the concept that the biological activity of therapeutic compounds is limited to unique and well-defined mechanisms. Contrarily, the simultaneous and combined use of active constituents, which is in line with the notion that nutrients are associated with the preservation of health status, supports the challenging search for novel therapeutic targets among bioactive food components (1). In this setting, polyphenols associate their intrinsic antioxidant and anti-inflammatory properties with a synergistic impact in metabolic functions and maintenance of cellular homeostasis (2). The intimate mechanism(s) is not completely understood but is probably associated with gene expression pathways that coordinate important intracellular signaling pathways (3, 4). One of the most important ways to maintain correct cell growth and differentiation is via the signal transduction cascade, activated by the binding of growth factors to their receptors. In particular, fibroblast growth factors (FGFs) are crucial in the regulation of tissue formation (5), and the potential effects of altered interactions between FGFs and their receptors (FGFRs) are attractive targets to be explored in the relationship between food intake and disease (6).

In humans, the binding of growth factors to specific receptors results in a pleiotropic response derived from the distinct tissue distribution and the complexity of these families of proteins. However, their immediate action comes from the activation and/or dysfunction of phosphorylation events in important signaling pathways. Dietary polyphenols are probable candidates to modulate and regulate disturbances in these pathways through relevant key molecules, including phospholipase C gamma (PLCγ) (7), the signal transducer and activator of transcription (STAT) protein (8), phosphoinositol-3 kinase/protein kinase B (PI-3K/AKT) (9), and rat sarcoma protein/mitogen activated protein kinase (RAS/MAPK [ERK-1/-2]) (10). It is therefore expected that human nutrition and growth factors are associated with inflammatory and metabolic disturbances (11–14). Moreover, point mutations in FGFRs affecting the function of FGFs-activated receptors have been shown to be consistently involved in cancer (15–17), atherosclerosis (18), and dysfunctions in growth and development (19, 20).

For the purpose of this study, we highlight the fact that unambiguous functional mutations in FGFR3 are involved in several diseases (21, 22), including skeletal dysplasia and osteoarthritis (23, 24). Because dietary polyphenols block the actions of some cytokines and FGFs that lead to cartilage degradation (25), we have tested the hypothesis that dietary polyphenols might have an important regulatory effect on the functional binding of FGFs to their receptors. We used murine chondrocytes to transduce a human mutated FGFR3 by substituting glycine with arginine at position 380 (G380R), which causes achondroplasia. When the receptor is activated by its specific ligand (FGF9), this mutation produces severe changes in cellular growth. We have previously shown (26–28) that this model is sensitive for gaining insight into the management of diseases associated with dysfunctions in cartilage. To reduce the complexities found in the composition of plant foods, but preserving the multiple and synergistic effects provided by the combination of multiple compounds, we have employed several fully characterized, polyphenol-rich plant extracts. The hypothesis is relevant because repression of mutated FGFR3 might illustrate the potential reversibility of a genetic condition and the feasibility of alternative therapeutic strategies (29, 30). Our findings support the view that the dietary incorporation of supplementary nutrients may be safely utilized to regulate intracellular signaling networks in the management of degraded cartilage phenotypes.

## Material and methods

### Chemical and reagents

Tetracycline, minimum essential medium alpha (α-MEM), heat-inactivated fetal bovine serum, and antibiotics (penicillin, Geneticin, streptomycin, and hygromycin) were obtained from Invitrogen (Carlsbad, CA, USA). Tris base, NaCl, NP-40, sodium deoxycholate, SDS, TWEEN-20, BSA, phenylmethylsulfonyl fluoride (PMSF), sodium fluoride (NaF), sodium orthovanadate (Na_3_VO_4_), aprotinin, pepstatin, leupeptin, and human recombinant FGF9 were obtained from Sigma (St. Louis, MO, USA). Antibodies against phospho-ERK-1/-2, ERK-1/-2, and GAPDH as well as horseradish peroxidase-conjugated goat anti-mouse IgG were purchased from Santa Cruz Biotechnology (Santa Cruz, CA, USA).

### Plant extracts and phenolic composition

We assayed different combinations of polyphenols in extracts prepared at a final concentration of 100 µg/mL in water or dimethyl sulfoxide from *Aspalathus linearis* (family Fabaceae; rooibos leaves), *Vitis vinifera* (family Vitaceae; grape seeds), *Citrus aurantium* (family Rutaceae; bitter orange), *Lippia citriodora* (family Verbenaceae; lemon verbena leaves), *Olea europaea* (family Oleaceae; olive leaves), and *Hibiscus sabdariffa* (family Malvaceae; karkadé). The criteria for inclusion were as follows: previous use in our laboratory in cell or animal models, commercialization (usually to prepare beverages), chemical characterization, and safety for human consumption. All extracts are readily available (Monteloeder, Elche, Spain) and information on the composition is provided in Supplementary Tables 1 and 2. Chromatographic methods, along with any additional extended data and references unique to these sections, are available as supplementary information in the online version. Of note, we also tested extracts from *Hypoxis rooperi* (family Hypoxidaceae; African potato) to ensure the lack of effects from lignans (data not shown). Because of distinct and significant effects obtained from *H. sabdariffa*, we only present a full report of data obtained with this mixture and a new preparation to concentrate its phenolic compounds. This concentration was performed via a described procedure that eliminates the residual presence of sugar, fiber, and other material, significantly reducing the concentration of organic acids and decreasing the concentration of prodelphinidin B3 (31) (Supplementary Table 2). This concentrated extract was employed in subsequent experiments to provide a concentration of compounds similar to that after the addition of normal extract (10 µg/mL in the culture medium).

### Cell culture and assessment of cell size and morphology

RCJ3.1C5.18 cells obtained from a mesenchymal cell line that differentiates into chondrocytes were used to test whether polyphenols modulate the FGF system. Cells were stably transfected with full-length human wild type (RCJ-FGFR3-WT) cells or with the G380R mutant FGFR3 (RCJ-FGFR3-G380R) cells included in a vector that overexpresses the receptor in absence of tetracycline, following procedures previously described (32, 33). Mutated FGFR3 (G380R) results in the overexpression of FGFR3, and in this model FGF9 provokes a reduction in cell growth and aberrant morphological changes in chondrocytes. The required materials were obtained from ProChon Biotech (Woburn, MA, USA). Cells were incubated at 37°C with 5% CO_2_ in the standard α-MEM culture medium supplemented with 15% heat-inactivated fetal bovine serum and antibiotics (600 µg/mL Geneticin, 100 U/mL penicillin/streptomycin, and 50 µg/mL hygromycin) with or without 2 µg/mL tetracycline. Results were provided by six independent experiments, in triplicate, and trypan blue was employed to detect viable, unstained cells. Cell size and morphology were monitored microscopically, as previously described (28).

### Chloride efflux measurements

To explore the effects of FGF9 in morphologic changes, we first measured the FGFR3-regulated chloride flux to assess the role of voltage gated chloride channels in chondrogenesis (34). This method was employed as a screening procedure to assess the potential benefits provided by polyphenols. In brief, measurements were made using N-[ethoxycarbonylmethyl]-6-methoxy-quinolinium bromide (MQAE) as a fluorescent indicator (35). MQAE has a high sensitivity to chloride, and changes in fluorescence inversely reflect changes in intracellular chloride concentration. Thus, a decrease in intracellular fluorescence indicates the conservation of or an increase in the intracellular chloride concentration. In the same way, an increase in fluorescence indicates a decrease in the intracellular chloride concentration. In both cases, a significant movement of water is produced with a consequent change in the voltage of the membrane (36). As previously published (28), measurements were made employing the appropriate controls, and the final concentration of FGF9 was set at 25 ng/mL according to dose–response experiments. Cells were grown to confluence in 96-well plates and loaded overnight with 0.8 mM MQAE at 37°C. Then, the medium was removed, washed, and incubated with chloride-containing buffer (to induce chloride channel activation) for 10 min at 37°C. The buffer was then removed and replaced by 100 µL of chloride-free buffer containing the desired concentration of FGF9.

### Assessment of ERK-1/-2 phosphorylation

Signals evoked by ligand-receptor interactions control fundamental cellular processes in chondrocytes. The experiments were focused to assess the previously unexplored effects of *H. sabdariffa* on the ERK-1 and -2 components of the MAPK cascade. ERK-1/-2 activation would represent an induction of chondrocyte maturation and, conversely, inhibition of ERK-1/-2 would suggest a blockade of maturation and cell death (37). Cells were lysed in a buffer with the following composition: 50 mM Tris–HCl pH 8.0, 150 mM NaCl, 1% NP-40, 0.5% sodium deoxycholate, 0.1% SDS, 1 mM PMSF, 1 mM NaF, 2 mM Na_3_VO_4_, 10 µg/mL aprotinin, 5 µg/mL pepstatin, and 10 µg/mL leupeptin. After centrifugation at 13,000 g for 20 min at 4°C, protein concentration was determined by the Bio-Rad protein assay (Bio-Rad laboratories, Hercules, CA, USA). Proteins from each sample (45 µg) were subjected to 10% SDS-polyacrylamide gels and were transferred to nitrocellulose membranes (Amersham Pharmacia Biotech, Buckinghamshire, UK). Membranes were then blocked and incubated overnight with the primary antibody in TBS containing 5% skim milk and 0.1% TWEEN-20. After washing, blots were incubated with horseradish peroxidase-conjugated goat anti-mouse IgG secondary antibody (1:2,000). Development was performed utilizing the ECL system (Amersham Pharmacia Biotech). Films were scanned and analyzed using a Kodak GL 200 Imaging System with Kodak Molecular Imaging Software (Rochester, NY, USA). Data are the result of six independent experiments, in triplicate, and the overall procedure was assessed using GAPDH as control.

### Measurement of extracellular matrix secretion

The extracellular matrix (ECM) provides mechanical support and a spatial context for signaling events by various cell surface growth factor receptors (38). We studied the effects of *H. sabdariffa* extract and concentrated polyphenols in cartilage matrix deposition in chondrocytes with a method based on Alcian blue staining (39). In brief, reagents and protocols were obtained from Lifeline Cell Technology (Frederick, MD, USA) and PromoCell (Heidelberg, Germany). Cells were seeded at a density of 2×10^5^ cells/well in six-well dishes, and differentiation was induced by adding 10 mM β-glycerophosphate and 50 µg/mL ascorbic acid to the medium. Cultures were fed with supplemented media every 2 days; FGF9 (25 ng/mL) was added to fresh growth medium, alone or in combination with bioactive compounds. Under these conditions, the amount of cartilage matrix (proteoglycan synthesis) was measured at 3, 7, and 10 days of culture (39). At these time points, cells were washed with PBS and stained with Alcian blue stain (1% in 3% acetic acid) for 30 min, washed again three times for 2 min in 3% acetic acid, and rinsed with distilled water. Solubility was achieved with 1% SDS, 1 h at 90°C, to measure the absorbance at 605 nm.

### Statistical analysis

All data are presented as the mean±SD. To test general differences among means, we employed ANOVA, followed by the Tukey-Kramer post-hoc test. To test specific differences between means, homogenous variances were assumed by the F-test, and Student's t-test (two-sided) was employed. *P*<0.05 was considered to be statistically significant. Graphics were prepared with GraphPad Prism 6 (La Jolla, CA, USA) in combination with the statistical software SPSS version 19 (Chicago, IL, USA).

## Results and discussion

**Fig. 1:**
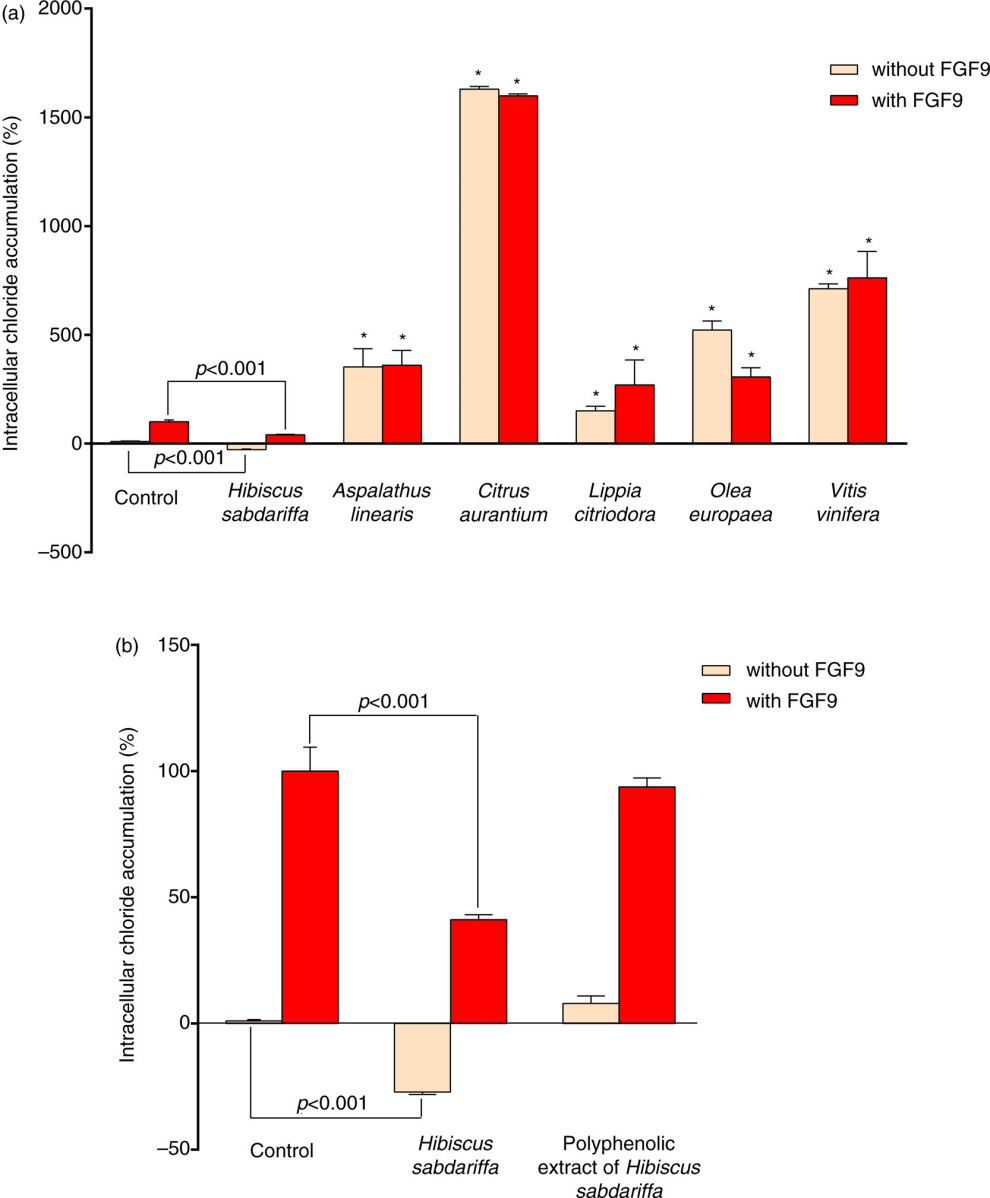
Intracellular chloride accumulation is modulated by polyphenols. Measurements are indicated as percentages with respect to values obtained in wild type cells and the effect of the addition of FGF9 (25 ng/mL; 100%) or the respective controls without FGF9 (0%). Data are expressed as the mean±SD (*n*=6–9 independent experiments in triplicate), indicating the relative increase or decrease in chloride concentration. (a) Among the different combinations of polyphenols, only those provided by *Hibiscus sabdariffa* decreased intracellular chloride accumulation. In contrast, all other combinations favored the accumulation of chloride. We decided to exclude these extracts in further analyses, and *p* values were provided only for *H. Sabdariffa*. (b) We concentrated this extract to individualize the effect of polyphenols, assessing any potential contribution of residual compounds and organic acids, and the values were compared with those obtained from the initial *H. sabdariffa* extract. Asterisks denote significant effects with respect to controls.

**Fig. 2:**
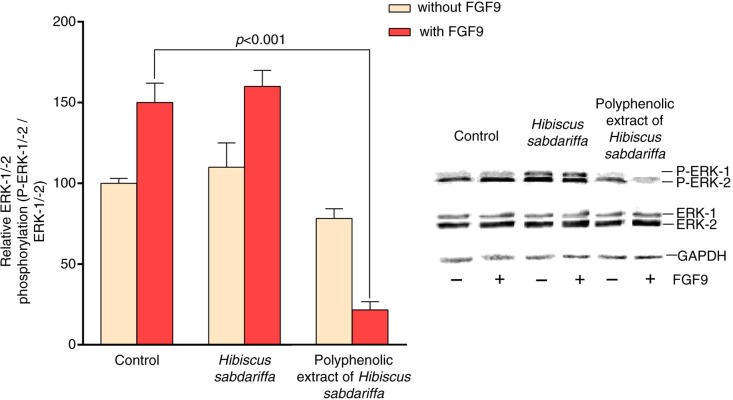
Relative ERK-1/-2 phosphorylation. Experiments were performed in FGFR3 (G380R) mutant RCJ3.1C5.18 chondrocytes with or without FGF9 (25 ng/mL) and further incubated with *Hibiscus sabdariffa* or its polyphenolic extract. Data were combined and expressed as the mean±SD (*n*=6, in triplicate). Representative results were included for illustrative purposes.

The expression in chondrocytes of the FGFR3 (G380R) mutation is currently considered to be the likely cause of the human chondrodysplasia, termed *achondroplasia* (40, 41). In these patients, there is an over-activation of FGFR3 in the presence of FGF9, which results in toxic effects in chondrocytes, preventing endochondral bone formation. Because this represents a gain-of-function mutation and FGFR3 is a transmembrane receptor tyrosine kinase that initiates signal transduction to the nucleus, it is reasonable to predict that successful therapeutic strategies would decrease or eliminate FGFR3 signals. Using mouse chondrocytes containing the human mutant FGFR3, it has been possible to test the effects of polyphenols and how these and other plant dietary compounds affect intracellular signaling networks in an achondroplastic model (2). To follow this approach, we measured the effect of commercially available extracts commonly used in edible preparations on the modulation of chloride efflux by FGFR3 (G380R) and FGF9. The arrangement of FGF9 and FGFR3 in this model induced an enlarged achondroplastic chondrocyte size and an increase in the intracellular chloride, which is likely the result of an inhibition of the chloride efflux (42). Intriguingly, most polyphenol combinations further increased the intracellular chloride concentration and potentiated the deleterious effects in chondrocytes (Fig. 1a). Quantitative effects, however, differed considerably, and we noted significant changes in cell size and a disproportionate 15-fold increase in intracellular chloride concentration caused by *C. aurantium*. In response to the reproducibility of these data, we excluded these dietary extracts in this study, but this information will drive studies to further investigate the role of resting membrane potential in the normal development of chondrocytes. Conversely, compounds from *H. sabdariffa* restrained the increase in the intracellular chloride concentration (Fig. 1b). Interpretation is difficult because data could indicate combined actions on FGFR3 signaling, binding of FGF9, or direct preservation of the membrane potential. Furthermore, analysis of the composition of the complex mixture of compounds in the initial extract revealed that a significant amount of organic acids and other materials were present together with polyphenols. We then eliminated these residual components to obtain an extract in which polyphenols were concentrated. Subsequent experiments, once corrected for the total amount of compounds, indicated that the effect of polyphenols in the regulation of chloride efflux, although significant, was less intense than that observed in the initial extract (Fig. 1b and Supplementary Tables 1 and 2). Contrary to what we expected, the results suggest a major role for organic acids, particularly hydroxycitric acid, but attribution of the effect to a single component is highly unlikely. Moreover, when we utilized both mixtures derived from *H. sabdariffa*, polyphenols appeared to be the compounds causing the inhibition of ERK-1/-2 (p42 and p44) phosphorylation. ERKs are important in cellular growth and development (17, 43–48), and the effects were detectable in cultures without FGF9, but statistical significance was only achieved in the presence of the ligand (Fig. 2). This effect is relevant because ERK-1/-2 phosphorylation was activated in FGFR3 (G380R) chondrocytes challenged with FGF9, confirming our previous findings (28). The role of the original *H. sabdariffa* extract may not be inferred from our data because it had no significant effect on the level of phosphorylation with or without FGF9, but it has been shown to target other multiple components (49–54). However, we limited the exploration to the MAPK pathway because ERK-1/-2 inhibition under these conditions is probably restricted to chondrocytes, in which this signaling system regulates cell growth, proliferation, and ECM accumulation (55–62).

**Fig. 3:**
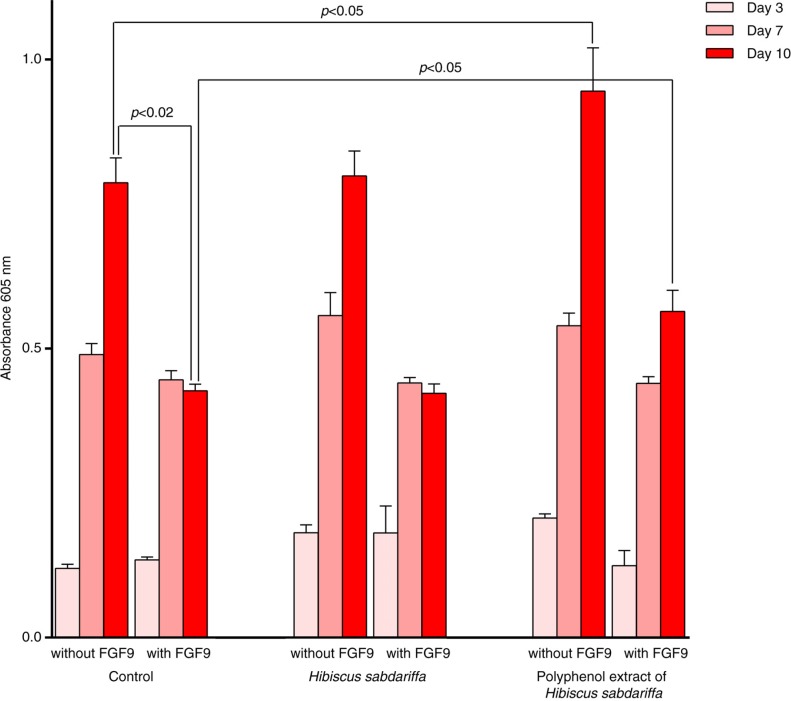
Extracellular matrix (ECM) deposition. Data were combined and expressed as the mean±SD (*n*=6, in triplicate), and the values indicate the amount of enrichment in the ECM utilizing Alcian blue staining of cells. Measurements were sequentially performed at days 3, 7, and 10 and included untreated cells (control) and activated cells in the presence of *Hibiscus sabdariffa* extract or its derived polyphenolic extract with or without FGF9 (25 ng/mL).

The stimulation by FGF9 of mutated FGFR3 also results in a dramatic decrease in ECM production in chondrocytes, an action that was reversed by *H. sabdariffa*. The effect was ostensible even in the absence of FGF9 and reached statistical significance after 10 days of treatment (Fig. 3). Total values were lower than those observed in wild type cells, indicating the probable contribution of other mechanisms, but active studies suggest that the differences are reduced with longer exposure time and higher concentrations (data not shown). ECM enrichment improves chondrocyte physiology due to the optimization of the signaling microenvironment of FGFs (63, 64). This effect is relevant in this environment because the formation of molecular complexes between growth factors and ECM proteins regulates dynamic interactions between proteins, bioavailability and function of many growth factors, and the modulation of FGFR signaling (65–67). It is therefore plausible that, in skeletal dysplasia and in diseases with prominently degraded cartilage, FGF9/FGFR3 modulators could prevent cartilage degeneration and/or promote cartilage regeneration (68, 69).

**Fig. 4:**
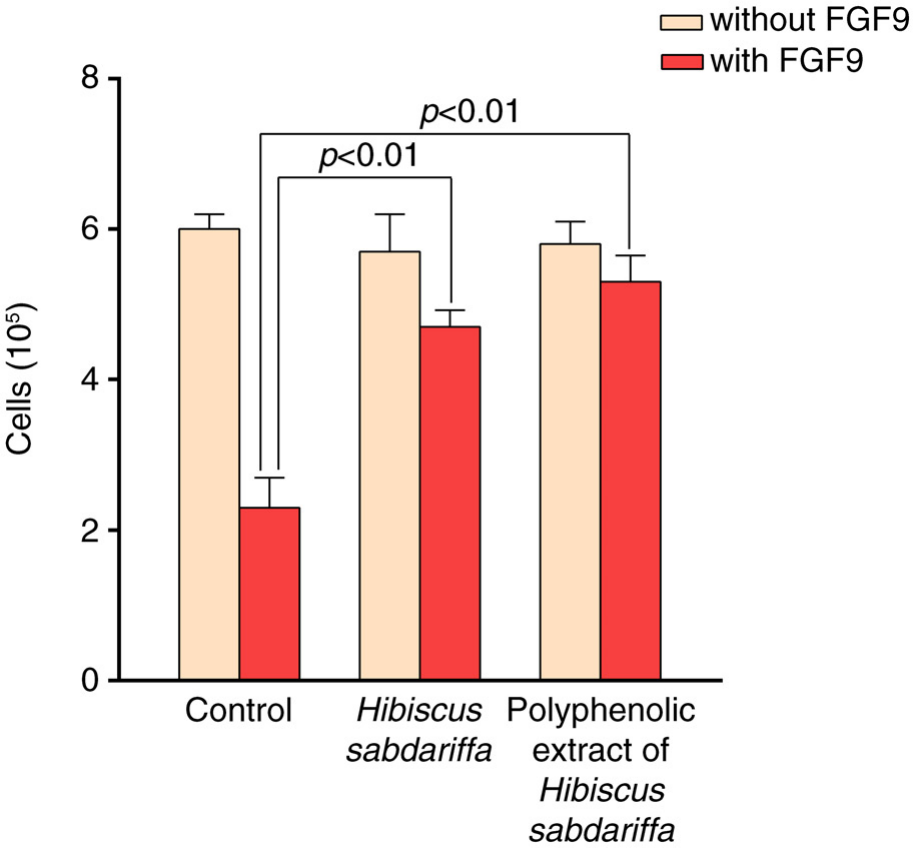
Chondrocyte proliferation comparing untreated cells (control) and treated cells with *Hibiscus sabdariffa* and its polyphenolic extract with or without FGF9 (25 ng/mL). Cell viability was determined by the trypan blue method. Data are expressed as mean±SD (*n*=6, in triplicate).

Accordingly, chondrocyte viability is low when cells bear the (G380R) mutation of FGFR3, the likely consequence of increased maturation and early cell death (70, 71). The effect is highly dependent on the presence of the ligand (FGF9), indicating the importance of active growth factors in the vicinity. In this model, FGF9 decreased cell survival in a few hours. *H. sabdariffa* treatment completely reversed the action of FGF9, and cell proliferation was similar to that observed in controls (Fig. 4), indicating the rescue of the FGFR3 (G380R) phenotype. Experiments should follow to ascertain whether individual compounds may elicit similar beneficial effects. However, safe concentrations are more difficult to achieve, and observations in animal models suggest that the synergistic action of natural compounds is a more likely possibility (3, 31, 55). Stronger evidence could be provided by the development of better *in vivo* and *in vitro* models employing human cells. It should be mentioned that recent cell reprogramming technologies might be utilized to obtain induced pluripotent stem cells differentiated into mutant chondrocytes that may confirm the anabolic effects we have found in chondrocytes treated with bioactive compounds from *H. sabdariffa*. As preliminary results, immunoprecipitation experiments of FGFR3 suggest that these bioactive compounds are not affecting the receptor directly. Thus, the exploration of related signaling pathways other than ERK-1/-2 in chondrocytes and the effect of some available drugs might also contribute to fully uncovering the actual molecular mechanisms (72–74). Perhaps it is time to contemplate significant dietary modifications in humans by food design and supplementation to obtain health benefits (75).

## Conclusion

**Fig. 5:**
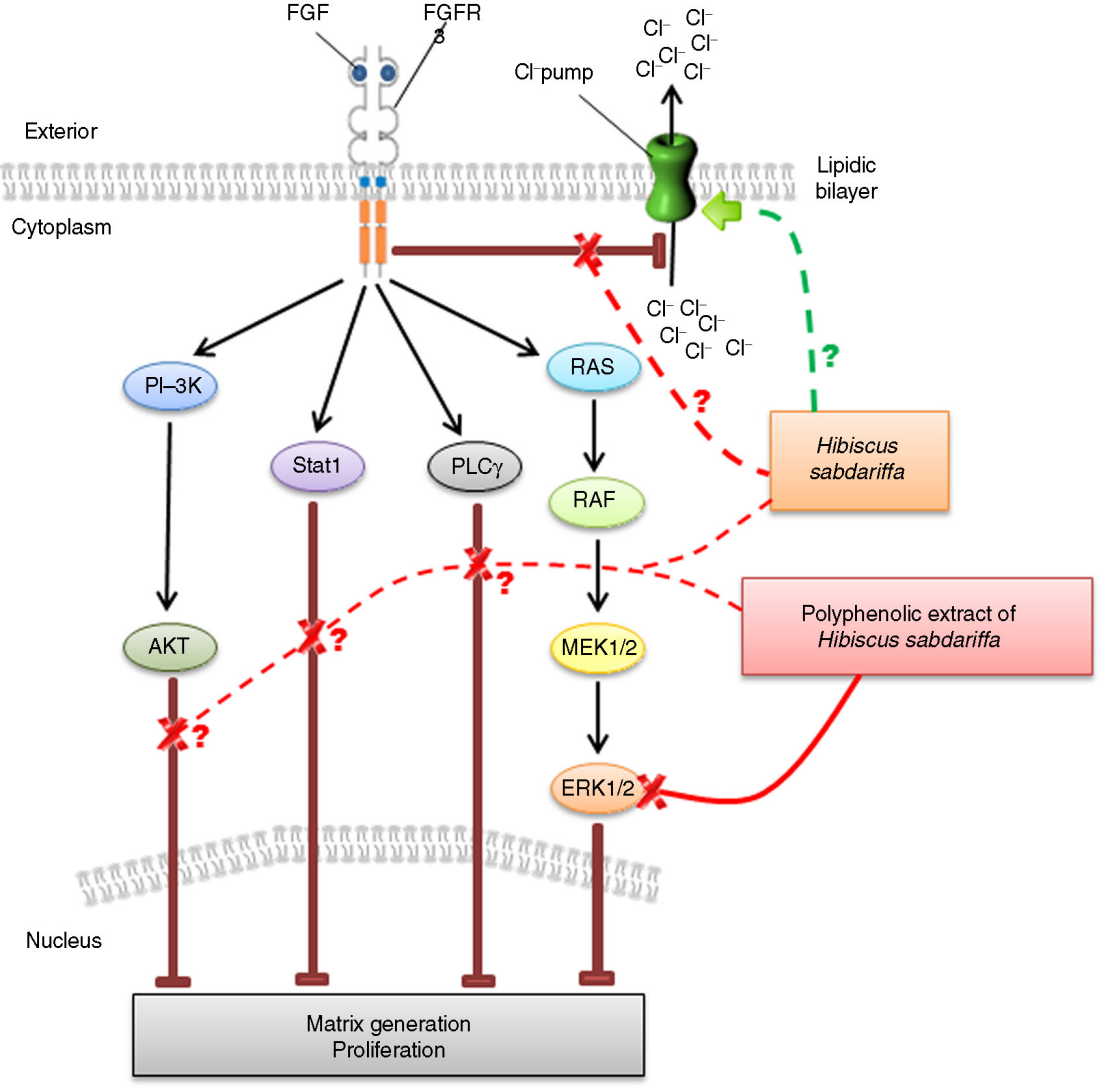
Illustrative summary of observed and proposed effects in the mutant chondrocyte model. Polyphenols target multiple components that promote and maintain tissue homeostasis beyond their anti-inflammatory and antioxidant effects, and their actions might be extended to disease prevention and intervention. Binding of fibroblast growth factor (FGF9) to its receptor (FGFR3) activates several important signaling pathways that are associated with the overall response to inflammation and are crucial in chondrocytes to regulate cell proliferation and extracellular matrix (ECM) generation. From the data, we consider the possibility that the action of complete *Hibiscus sabdariffa* extract might be different from that observed with concentrated polyphenols. In particular, polyphenols appear more active in the inhibition of ERK-1/-2 phosphorylation and in the increase of ECM generation, whereas the combination of organic acids and polyphenols could be more active in reversing the intracellular chloride accumulation and increasing cellular proliferation. *H. sabdariffa* reverses the toxic actions of the FGF9/FGFR3 interaction in mutated chondrocytes. The results indicate the need for a search for human models to fully establish actual mechanisms as well as the potential modification of diets to achieve human health benefits. AKT (PKB): protein kinase B; ERK 1/2 (MAPK): extracellular signal-regulated kinases 1 and 2; FGF: fibroblast growth factor; FGFR: fibroblast growth factor receptor; MEK1/2: MAPK kinases 1 and 2; PI-3K: phosphatidylinositide 3-kinase; PLCγ: phospholipase C gamma; RAF: rapidly accelerated fibrosarcoma (serine/threonine-protein kinase); RAS: rat sarcoma protein (GTPase); STAT1: signal transducer and activator of transcription 1.

With the rationale that some nutrients with intrinsic anti-inflammatory and antioxidative effects may mitigate a deleterious response leading to degraded cartilage, we assayed the activity of dietary polyphenols in murine cells with the transduced human (G380R) mutation in FGFR3. Most polyphenols had no effect, but those provided by *H. sabdariffa* rescued chondrocytes from FGF9-induced toxic effects, supporting the view that plant-derived dietary products affect intracellular signaling networks and may be safely utilized in the management of degraded cartilage phenotypes. *H. sabdariffa* rescued mutated FGFR3 chondrocytes, restoring normal growth, decreasing the intracellular chloride concentration, inhibiting ERK-1/-2 phosphorylation, and increasing the generation of ECM (Fig. 5). Our data indicate the crucial role of growth factors in the survival of chondrocytes and suggest effective and applicable therapeutic strategies for patients with degraded cartilage. *H. sabdariffa* would have the advantage that it has been safely administered to large numbers of humans for millennia, including pregnant women and infants.

## Supplementary Material

The impact of polyphenols on chondrocyte growth and survival: a preliminary report
