# Supplementary material for: The impact of polyphenols on chondrocyte growth and survival: a preliminary report
Source: Food Nutr Res. 2015 Oct 5;59:10.3402/fnr.v59.29311. doi: 10.3402/fnr.v59.29311 (PMC4595466; doi:10.3402/fnr.v59.29311)
Supplement: The impact of polyphenols on chondrocyte growth and survival: a preliminary report [file FNR-59-29311-s001.docx]

## Supporting information

**Action of polyphenols in bone formation**

**The impact of polyphenols in chondrocyte growth and survival: a preliminary report**

Salvador Fernández-Arroyo^1,2,*^, Fernando Huete-Toral^3^, María Jesús Pérez de Lara^3^, María de la Luz Cádiz-Gurrea^4,5^, Laurence Legeai-Mallet^6^, Vicente Micol^7^, Antonio Segura-Carretero^4,5^, Jorge Joven^1,2^, Jesús Pintor^3*^

^1^ Unitat de Recerca Biomèdica, Hospital Universitari de Sant Joan, IISPV, Universitat Rovira i Virgili, C/ Sant Joan s/n, 43201 Reus, Spain

^2^ Campus of international excellence southern Catalonia. C/ de l'Escorxador s/n, 43003 Tarragona, Spain

^3^ Department of Biochemistry, Faculty of Optics and Optometry. Universidad Complutense de Madrid, C/Arcos de Jalón 118, 28037 Madrid, Spain

^4^ Functional Food Research and Development Center, Health Science Technological Park, Avenida del Conocimiento s/n, E-18100 Armilla (Granada), Spain

^5^ Department of Analytical Chemistry, University of Granada, Spain

^6^ Imagine Institute – INSERM U1163. Necker Hospital for Sick Children, 24 Bd. Montparnasse, 75015 Paris, France

^7^ Institute of Molecular and Cell Biology, Miguel Hernández University, Avda. Universidad s/n, Elche 03202, Spain. CIBER (CB12/03/30038, CIBERobn, Instituto de Salud Carlos III)

* Corresponding authors:

Salvador Fernández-Arroyo.

Unitat de Recerca Biomèdica, Hospital Universitari de Sant Joan, IISPV, Universitat Rovira i Virgili, C/ Sant Joan s/n, 43201 Reus (Tarragona), Spain. E-mail: [sfernandez@fiispv.cat](mailto:sfernandez@fiispv.cat) Underlying research materials and raw data related to this manuscript are available on request from this author.

Jesús Pintor

Biochemistry Department, Faculty of Optics and Optometry. Universidad Complutense de Madrid, C/Arcos de Jalón 118, 28037 Madrid, Spain. E-mail: [jpintor@vet.ucm.es](mailto:jpintor@vet.ucm.es)

**Chromatographic methods to assess the composition of plant extracts.** Polyphenols and other polar compounds (e.g., organic acids or simple phenols) were characterised using high-performance liquid chromatography (HPLC) in a suitable apparatus equipped with a C18 reverse phase column coupled to mass spectrometry (MS) utilising an electrospray ionisation source (ESI). In particular, *Aspalathus linearis* (Family *Fabaceae*; rooibos leaves) was characterised utilising a time-of-flight (TOF) and an ion trap (IT) as mass spectrometers (1). To analyse *Lippia citriodora* (Family *Verbenaceae*; lemon verbena leaves), we employed capillary electrophoresis coupled to an ESI-TOF/IT-MS platform (2). *Olea europaea* (Family *Oleaceae*; olive) leaves were characterised utilising a quadrupole-TOF as mass spectrometer (HPLC-ESI-QTOF-MS) (3). *Vitis vinifera* (Family *Vitaceae*; grape) seeds were analysed using an HPLC system coupled to a photodiode-array detector (4). For *Citrus aurantium* (Family *Rutaceae*; bitter orange), the characterisation was conducted using a diode-array detector (DAD) (5). Finally, the phenolic and other polar compounds from *Hibiscus sabdariffa* (Family *Malvaceae*; karkade) were characterised utilising an HPLC-ESI-DAD-IT-MS (6) and quantitated with an HPLC-ESI-DAD-TOF-MS (7); the concentrated polyphenolic extract of *Hibiscus sabdariffa* was prepared as described (8).

**Table S1.** Qualitative composition of phenolic compounds in the assayed plant extracts

| ***Aspalathus***  ***linearis*** | ***Citrus***  ***aurantium*** | ***Lippia***  ***citriodora*** | ***Olea europaea***  **leaves** | ***Vitis vinifera***  **seeds** |
| --- | --- | --- | --- | --- |
| Aspalaninin  Aspalathin  Carlinoside (and derivatives)  Esculin  Iso-orientin  Isovitexin  Luteolin (and glycoside derivatives)  Nothofagin  Orientin  Patuletin-7-glucoside  Quercetin (and glycoside derivatives)  Secoisolariciresinol  Vicenin-2 | Catechin  Chlorogenic acid  Cinnamic acid (and derivatives)  Epicatechin  Ferulic acid  Gallic acid  Hydroxybenzoic acid  Naringin  *p*-Coumaric acid  Rosmarinic acid  Rutin  Syringic acid  Tyrosol  Vanillic acid | Acacetin-7-diglucuronide  Apigenin-7-diglucuronide  Campneoside I  Chrysoeriol-7-diglucuronide  Cistanoside F  Eukovoside  Forsythoside A  Gardoside  Luteolin-7-diglucuronide  Martinoside  Theveside  Verbascoside (and derivatives) | 7-epiloganin  Apigenin (and glycoside derivatives)  Elenolic acid derivatives  Hydroxybenzoic acid  Hydroxytyrosol  Ligstroside  Lucidumoside C  Luteolin (and glycoside derivatives)  Oleoside (and derivatives)  Oleuropein (and derivatives)  Rutin  Vanillin  Verbascoside | Caffeic acid  Catechin  Chlorogenic acid  Epicatechin  Epicatechin-gallate  Gallic acid  *p*-Coumaric acid  Procyanidin B1  Procyanidin B2  Procyanidin B3  Procyanidin B4  Protocatechuic acid  Quercetin  Rutin  Syringic acid  *t*-piceatannol  *t*-resveratrol  Vanillic acid |

| Compound | *Hibiscus sabdariffa* | Polyphenolic extract of *Hibiscus sabdariffa* |
| --- | --- | --- |
| Hydroxycitric acid | 0.8288 | - |
| Hibiscicus acid | 3.1122 | 1.2813 |
| Delphinidin-3-*O*-sambubioside | 0.2701 | 2.0732 |
| Cyanidin-3-*O*-sambubioside | 0.1939 | 0.8714 |
| Chlorogenic acid | 0.572 | 1.0647 |
| Hibiscus acid dimethyl ester | - | 0.0530 |
| Methyl digallate | - | 0.0280 |
| Myricetin-3-*O*-arabinogalactose | 0.0057 | 0.0476 |
| Coumaroylquinic acid | - | 0.0077 |
| Quercetin-3-*O*-sambubioside | 0.0304 | 0.0767 |
| Quercetin-3-*O*-rutinoside | 0.0495 | 0.0495 |
| 5-*O*-Caffeoylshikimic acid | 0.0172 | 0.0353 |
| Kaempferol-3-*O*-sambubioside | - | 0.0112 |
| Quercetin-3-*O*-glucoside | 0.0144 | 0.0307 |
| Kaempferol-3-*O*-rutinoside | 0.0092 | 0.0219 |
| Methyl epigallocatechin | - | 0.0031 |
| Myricetin | - | 0.0477 |
| *N*-feruloyltyramine | 0.0099 | 0.0087 |
| Prodelphinidin B3 | 0.1839 | 0.0033 |
| Quercetin | 0.0121 | 0.0580 |

**Table S2**. Phenolic compounds characterised in *Hibiscus sabdariffa* and its polyphenolic extract. The values indicate the concentration (in μg/mL) of each compound in the culture medium. To provide the same total amount of compounds, we added 100 μg/mL of the *Hibiscus sabdariffa* extract or 10 μg/mL of the polyphenolic extract of *Hibiscus sabdariffa*.

**Additional references**

1. Iswaldi I, Arraez-Roman D, Rodriguez-Medina I, Beltran-Debon R, Joven J, Segura-Carretero A, et al. Identification of phenolic compounds in aqueous and ethanolic rooibos extracts (*Aspalathus linearis*) by HPLC-ESI-MS (TOF/IT). Anal Bioanal Chem. 2011;400:3643-54.

2. Quirantes-Pine R, Arraez-Roman D, Segura-Carretero A, Fernandez-Gutierrez A. Characterization of phenolic and other polar compounds in a lemon verbena extract by capillary electrophoresis-electrospray ionization-mass spectrometry. J Sep Sci. 2010;33: 2818-27.

3. Quirantes-Pine R, Lozano-Sanchez J, Herrero M, Ibañez E, Segura-Carretero A, Fernandez-Gutierrez A. HPLC-ESI-QTOF-MS as a powerful analytical tool for characterising phenolic compounds in olive-leaf extracts. Phytochem Anal. 2013;24:213-23.

4. Doshi P, Adsule P, Banerjee K, Oulkar D. Phenolic compounds, antioxidant activity and insulinotropic effect of extracts prepared from grape (Vitis vinifera L) byproducts. J. Food Sci Technol. 2015;52:181-90.

5. Karoui IJ, Marzouk B. Characterization of bioactive compounds in tunisian bitter orange (*Citrus aurantium* L.) peel and juice and determination of their antioxidant activities. Biomed Res Int. 2013;Article ID 345415.

6. Rodriguez-Medina IC, Beltran-Debon R, Micol V, Alonso-Villaverde C, Joven J, Menendez JA, et al. Direct characterization of aqueous extract of *Hibiscus sabdariffa* using HPLC with diode array detection coupled to ESI and ion trap MS. J Sep Sci. 2009;32:3441-8.

7. Fernández-Arroyo S, Rodríguez-Medina IC, Beltrán-Debón R, Pasini F, Joven J, Micol V, et al. Quantification of the polyphenolic fraction and in vitro antioxidant and in vivo anti-hyperlipemic activities of *Hibiscus sabdariffa* aqueous extract. Food Res Int. 2011;44:1490-5.

8. Herranz-Lopez M, Fernandez-Arroyo S, Perez-Sanchez A, Barrajon-Catalan E, Beltran-Debon R, Menendez JA, et al. Synergism of plant-derived polyphenols in adipogenesis: Perspectives and implications. Phytomedicine 2012;19:253-61.
